# Weight Loss in Post-Chemoradiotherapy Head and Neck Cancer Patients

**DOI:** 10.3390/nu14030548

**Published:** 2022-01-27

**Authors:** Zalina Abu Zaid, May Kay Neoh, Zulfitri Azuan Mat Daud, Nor Baizura Md Yusop, Zuriati Ibrahim, Zuwariah Abdul Rahman, Norshariza Jamhuri, Aishah Zafirah Abdul Azim

**Affiliations:** 1 Department of Dietetics, Faculty of Medicine and Health Sciences, Universiti Putra Malaysia, Serdang 43400, Malaysia; dtneoh@nci.gov.my (M.K.N.); zulfitri@upm.edu.my (Z.A.M.D.); norbaizura@upm.edu.my (N.B.M.Y.); zuriatiib@upm.edu.my (Z.I.); dtshariza@nci.gov.my (N.J.); aishahzaf@upm.edu.my (A.Z.A.A.); 2 Department of Dietetics, Hospital Pengajar Universiti Putra Malaysia, Serdang 43400, Malaysia; 3 Department of Dietetic and Food Service, National Cancer Institute, Ministry of Health, 4, Jalan P7, Presint 7, Putrajaya 62250, Malaysia; dtzuwariah@nci.gov.my

**Keywords:** oncology, chemotherapy, radiation, weight loss

## Abstract

Background: Weight loss is well-known among head and neck (HNC) patients during radiotherapy and could continue after the treatment is completed. Weight monitoring is essential for treatment outcomes and cancer surveillance. The purpose of the study is to evaluate the weight loss during and post-treatment among HNC patients. Methods: A total of 45 out of 50 patients post-treatment were included in this secondary treatment. Data were collected at baseline, at the completion of radiotherapy and one month after completion of radiotherapy. Results: The mean weight loss was 4.53 ± 2.87 kg (7.4%) during treatment and 1.38 ± 2.65 kg (2.1%) post-treatment. There were significant improvements one month after completion of radiotherapy in Patient-Generated Subjective Global Assessment (PGSGA), muscle mass, nutrition impact symptoms (NIS) score, energy and protein from oral intake (*p* < 0.0001). Energy and protein from oral nutritional supplements (ONS) decreased significantly (*p* < 0.0001). Conclusions: The results of this study underline the importance of early identification and monitoring post-treatment in HNC patients. The post-treatment recovery stage is very important for HNC patients to ensure a healing process.

## 1. Introduction

Head and neck cancer (HNC) include tumors of the oral cavity, oropharynx, hypopharynx, and lymph nodes in the neck [1]. It is the sixth most common form of cancer worldwide, accounting for approximately 5% of new cancer cases in 2018 [2]. Many HNC patients are diagnosed at an advanced stage (Stage II/IV) and most of them suffer conditions of nutritional vulnerability with a high risk of malnutrition [3]. Tumor location directly impacts patients’ oral intake, taste, appetite, and weight loss [4].

Patients with HNC experience substantial weight loss (WL) during and after treatment [5]. More than 50% of HNC patients lose more than 5% of their body weight at the time of initial treatment [6]. Critical WL is defined when the body weight loss is >5% during RT [7]. Langius et al. (2016) [7] found that patients who had critical WL >5% prior to treatment are independently associated with a 1.7 times higher risk of mortality from HNC. In HNC patients at an advanced stage, WL could influence morbidity, which reduces the quality of life and treatment tolerance [8].

Current treatment of advanced HNC requires multimodality therapy such as surgery, radiotherapy (RT), concurrent chemoradiotherapy (CCRT), which have become a standard of care for HNC patients [9]. RT with or without chemotherapy in HNC cancer patients induces oral side effects such as mucositis, dry mouth, and thick saliva mucositis, decreased food intake, and WL in up to 80% of patients [5]. A study by Farhanghar et al. (2014) has shown that patients with multiple nutrition impact symptoms (NIS) or greater total symptom scores are more prone to have reduced dietary intake and WL [10]. Therefore, the total symptom burden is an important risk factor for WL in HNC patients [11].

A study by Ottosson et al. (2013) has shown that HNC patients have a rapid decrease in weight of about 11.3% during the acute phase, which changed or decreased at the five-month follow-up after completion of the RT [5]. Still, there is a lack of studies exploring HNC patients’ weight loss and nutritional situation over a longer period, i.e., from diagnosis, during and to the end of the treatment, and post-treatment. This information is essential for nutrition evaluation and for monitoring HNC patients to ensure the healing process is optimized. Hence, this study aimed to investigate changes in body weight and nutritional parameters in HNC patients receiving RT over time.

## 2. Materials and Methods

### 2.1. Study Design and Setting

This study was carried out as part of the previous prospective observational study on the changes in NIS, nutritional and functional status among HNC patients undergoing RT [12].

### 2.2. Participants

Participants in this analysis included 45 out of 50 patients who were recruited one month after completion of their treatment (post-treatment).

### 2.3. Data Collection

Study instrument used was an interview administered questionnaire. Assessment of socio-demographic, clinical characteristics, nutritional status consists of malnutrition status, anthropometric measurements and dietary intake, and nutrition impact symptoms were collected at baseline, end of treatment and one month after completion of RT. Detailed information about the study instruments was reported elsewhere [12].

### 2.4. Sample Size

The required sample size is 40 patients. Detailed information about the sample size calculation was reported elsewhere [12].

### 2.5. Ethical Approval

This study has obtained ethical approval, as described in detail elsewhere [12].

### 2.6. Statistical Analysis

For the statistical analysis, the IBM SPSS Statistics for Windows, Version 23.0. Armonk, NY: IBM Corp. was used. Shapiro–Wilk test was used to check for normality of the data. If the data were not normally distributed, the natural logarithm of the values was used. Continuous variables which are normally distributed are presented as mean and standard deviation while median and interquartile range is presented for continuous variables which are not normally distributed. The Mann–Whitney U-test was used to test the differences between groups for ordinal data. Changes of normally distributed continuous variables over time (body weight, muscle mass, total energy and protein intake) were used repeated-measures ANOVA. A Friedman test was used to analyze changes of abnormally distributed continuous variables (PGSGA score, fat mass, NIS score, and, ONS energy and protein intake). In the case of deviation from sphericity, a Greenhouse–Geisser correction for degrees of freedom was used. A statistical probability of *p* < 0.05 was considered significant.

## 3. Results

Table 1 described the socio-demographic characteristics, clinical characteristics, lifestyle habits and type of treatment of the HNC patients. The median age in the population was 60 years old and the age range was 21 to 78 years old. More than half (52%) of the patients were in the old adults’ category at ≥60 years old. The recruitment for this study has shown that there are more males than females with HNC (78% versus 22%). Twenty-one Malay (42%), nineteen Chinese (38%) and ten Indian (20%) patients participated in this study. There were 40% that had a lower education level, 68% were married, 28% were working. Half of the 50 patients were active smokers or ex-smokers. The majority of the patients were non-alcoholic with 76.5%. A total of 28% of them had a family history of cancer and 58% had pre-existing comorbidities. In this study, 84% were in stage III and IV, and over half of the HNC patients had nasopharynx cancer. In addition, seventeen patients received RT (34%) and thirty-three patients received CCRT (60%).

In this study, 50 HNC patients were able to complete RT treatment (7 weeks) and completed the data collection until the end of treatment. However, there were five HNC patients that defaulted on the follow-up after 1 month of treatment. Finally, the total number of HNC patients eligible for post-treatment analysis was 45 HNC patients.

Table 2 shows the changes in nutritional status and NIS at baseline, end and post-treatment. The mean weight loss was 4.53 ± 2.87 kg (7.4 ± 4.1%) during treatment and the median weight loss was 0.7 kg and a range of −3.9 to 0.6 kg (2.1 ± 4.7%) post-treatment. The body weight was 60.24 ± 14.73 kg at baseline of treatment and significantly declined to 55.71 ± 13.62 kg at the end of treatment with a further drop to 56.26 ± 1.8 kg at post-treatment (*p* < 0.0001). Fat mass loss was higher compared to muscle mass loss for both during and post-treatment. The PGSGA score showed a significant increase from baseline to end of treatment but was able to improve by post-treatment (*p* < 0.0001). The mean NIS score significantly increased from 21.78 ± 4.59 to 48.34 ± 8.79 at the end of treatment but improved to 27.08 ± 10.19 by post-treatment (*p* < 0.0001).

The total energy and total protein intake increased from baseline until post-treatment but there were no significant changes for both results. Nevertheless, there were significant changes in oral food energy, oral food protein, ONS energy and ONS protein intakes. The oral food energy and protein intakes declined from baseline to end of treatment but improved again at post-treatment, while the ONS energy and protein intakes increased from baseline to end of treatment but dropped back at post-treatment (*p* < 0.0001).

**Figure 1:**
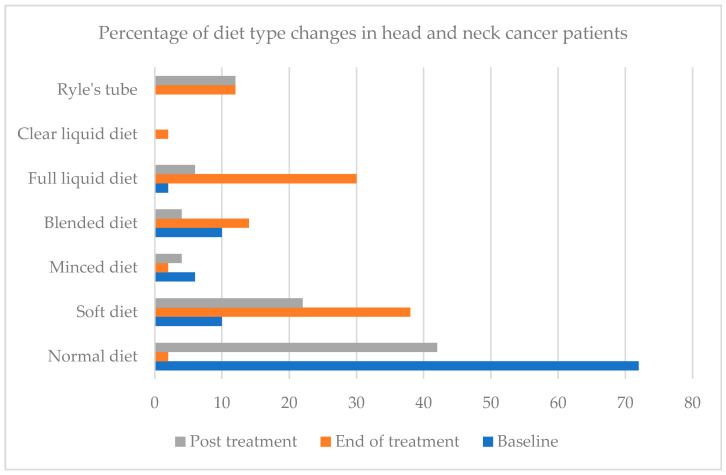
Changes of diet type at baseline, end and post-treatment of head and neck cancer patients.

The percentage of diet-type changes in head and neck cancer patients at baseline, end and post-treatment is presented in Figure 1. The majority of the patients were on a normal diet (72%) at baseline and decreased to 2% by the end of treatment but increased back to 42% at post-treatment. A soft diet was gradually increased from 10% to 38% by the end of treatment but dropped back to 22% at post-treatment. At the same time, there was an increasing trend for a blended diet, full liquid diet and clear liquid diet at the end of the treatment. A total of 12% of HNC patients on Ryle’s tube feeding at the end of treatment remain here at post-treatment. HNC patients who were on liquid textures (blended diet, full liquid diet, clear liquid, Ryle’s tube feeding) was at 58% at end of treatment and 68% at post-treatment while HNC patients who were on a solid or semi-solid diet (normal diet, soft diet, minced diet) was 42% at the end of treatment and 32% at post-treatment.

At the beginning of treatment, the majority of HNC patients (90%) did not receive any oral nutritional supplement (ONS). However, 96% of HNC patients were on ONS at end of treatment and 80% of HNC patients at post-treatment (Table 3).

Over half of the patients with HNC had difficulty chewing symptoms and 38% of them had a loss of appetite and dry mouth symptoms before treatment (Table 4). At the end of treatment, all 50 HNC patients experienced dry mouth and taste changes and more than 80% of them experienced sore mouth, lack of energy, loss of appetite, difficulty swallowing and chewing (Table 4). While post-treatment, all of the HNC patients had improved NIS.

As presented in Table 5, HNC patients had the highest NIS interference score of taste changes and loss of appetite, followed by swallowing and chewing difficulty, dry mouth, pain, sore mouth and thick saliva at the end of treatment. More than half of HNC patients still experienced taste changes, difficulty chewing, loss of appetite, dry mouth and thick saliva at post-treatment but had improving NIS interference scores.

## 4. Discussion

The patients in this study started to lose weight significantly during treatment and had further declined body weight until post-treatment (Table 2). With the pre-treatment weight or weight on the first week of radiotherapy as a baseline, the mean percentage of weight loss was already considerable after 2 to 3 weeks of radiotherapy and the weight loss at the end of radiotherapy was even worse. After treatment, weight was monitored and documented and approximately a quarter of the patients had regained weight. It could have been expected that during this period, which might be called rehabilitation, patients should have regained the lost weight. Percentage of weight loss is considered an easy, clinically relevant, simple, and reliable predictor for malnutrition. Involuntary 5% weight loss in less than 1 month without stabilization or beginning weight gain was shown to be a valid marker for malnutrition in HNC patients. Langius et al. (2016) [7] found that patients who experience weight loss of >5% within 8 weeks of RT was significant, and was a sign of compromised nutritional status. This was shown to increase the rate of complications, which can lead to high mortality rates and reduce the quality of life.

All the HNC patients were malnourished with 32% being moderately malnourished and 68% being severely malnourished at the end of treatment. This is comparable with Wei’s study that showed a strong correlation between involuntary weight loss and PG-SGA, where a PG-SGA score difference had a strong positive relationship with the percentage of weight loss at the end of treatment [13]. Malnutrition at the time of diagnosis can negatively affect clinical decisions of treatment options for patients due to unpredictable complications and curative rates in the management of cancer. In addition, it can cause treatment interruption that affects the effectiveness of treatment, high infections risk, delay wound healing, increase the length of hospital stay and increase morbidity and mortality [14]. After a month of treatment, 42% of the patients improved back to well-nourished as they recovered from the side effects of the treatment.

The majority of HNC patients were at an advanced stage and were found to have a high tendency for nutritional depletion with inadequate energy and protein intake. The average daily energy and protein intake in this study were below the ESPEN recommended guideline of 25–30 kcal/kcal of body weight and 1.2–1.6 g/kg/body weight at baseline of treatment [15]. An improvement of average daily energy and protein intake was found at the end and after the treatment compared to the baseline (Table 2), however, the intake was still below the ESPEN recommendation, respectively. The patients in this study who started to lose weight continued to lose it throughout the treatment as a consequence of inadequate energy and protein intake. As the treatment was completed, the average of calories and protein intake increased, as compared to the baseline intake which is similar to another study [16]. It was expected that significant weight loss is due to eating problems. Some studies have shown a relation of insufficient knowledge of food and nutrition among cancer patients, which has an impact on nutritional outcomes [17]. Some studies have shown that the majority of cancer patients who have dietary perceptions and beliefs with specific food restrictions, result in a high risk of malnutrition after diagnosis [18,19].

Most HNC patients were required to change their type of diet during treatment which was similar to [5,16] where some HNC patients could not eat and needed to adjust their food intake or rely on nutritional support to get sufficient energy and protein intake (Figure 1). As patients suffer multiple NIS in the middle and at the end of treatment, nearly 50% of patients chose a liquid diet rather than a normal diet. For those who are suffering from the symptom of a dry mouth, foods that were cold, moist, softly textured, and came with sauces, broths or soupy foods were preferred. In the study, dry foods and spices were required to be excluded due to a sore mouth or throat, which showed that a normal diet was not tolerated among HNC patients after treatment. Many HNC patients had struggled to eat sufficiently as they lost the pleasure in food and eating due to changes in taste and limited food choices [20].

The majority of HNC patients in this study required ONS, which is almost similar to Hopanci et al.’s (2017) [21] study in which 95% of the HNC patients were prescribed ONS during RT as it was shown that during treatment, fat mass and fat-free mass remain unchanged (Table 3). In this study, about 70% of total energy and protein intake were contributed to ONS in the middle and end of treatment with the majority of patients having shown a high score of NIS that interrupted their dietary intake and lead to weight loss.

The NIS score and ONS energy and protein intake showed an increasing trend during treatment. The majority of patients continued to have eating problems and causes of eating problems such as pain, chewing and swallowing difficulties, loss of appetite, dry mouth, taste changes, sore mouth, and thick saliva required ONS to increase their energy and protein intake. While in the post-treatment, as it is a rehabilitation phase, the persistence of NIS is reduced meaning oral intake should have improved, which led to less total energy and protein from ONS.

All HNC patients had multiple NIS at the end of treatment, resulting in a high NIS score (Table 4), which was comparable with Kubrak et al.’s (2013) study [22]. All of these NIS were interrelated with each other in that they interfered with dietary intake. Dry mouth exposes a higher risk of oral mucositis due to the breakdown of the mucosal barrier that promotes bacterial growth with high levels of pro-inflammatory cytokines, which may cause anorexia problems. Multiple NIS are associated with malnutrition risk, weight loss, dietary intake and quality of life. The majority of patients in the present study continued to have taste changes, chewing difficulties, mouth dryness and thick saliva after completion of the treatment. These NIS were the most common side effects after 1 and 6 months.

The NIS data and malnutrition status in this study allow us to design a more effective nutrition intervention and monitor the patients’ condition in the future. This is the first study to evaluate the post-treatment RT among HNC patients. It is crucial to generate comprehensive data of the post-treatment RT assessment in order to identify early symptoms, prevent severe weight loss and improve treatment outcomes by providing an effective nutritional intervention among HNC patients. This may highlight the need for continuous assessment of malnutrition status, dietary changes, and NIS to provide comprehensive nutritional intervention and monitoring after completion of treatment. A limitation of this study should be noted. The generalization of these results among HNC patients may not be allowed as the sample size is small. Nevertheless, the result of this study serves as a reference and benchmark for further research on weight changes and other nutrition parameters in HNC during and after post-treatment RT, to ensure the optimization of nutritional intervention.

## 5. Conclusions

Overall, HNC patients in this study experienced weight loss, a decline of energy and protein intake, and NIS scores at the end of treatment but all of these parameters were improved during post-treatment. The post-treatment recovery stage is also very important for HNC patients as eating problems still remain a common issue and food intake adjustment is required from time to time to ensure a healing process. Continuous follow-up is therefore important to monitor the progression of complications and for the maintenance of nutritional status.

## Figures and Tables

**Table 1:** Patient socio-demographics, clinical characteristics, lifestyle habits and types of treatment.

Characteristics	Overall (*n* = 50)
Age (years), median (IQR)	60 (49–67)
Age groups, *n* (%)	
<40 years old	6 (12)
40–59 years old	18 (36)
≥60 years old	26 (52)
Gender, *n* (%)	
Male	39 (78)
Female	11 (22)
Race, *n* (%)	
Malay	21 (42)
Chinese	19 (38)
India	10 (20)
Education level, *n* (%)	
Primary or below	20 (40)
Secondary or above	30 (60)
Marital status, *n* (%)	
Single	13 (26)
Married	37 (74)
Working status, *n* (%)	
Yes	14 (28)
No	36 (72)
Co-morbidities, *n* (%)	
Yes	29 (58)
No	21 (42)
Smoking history, *n* (%)	
Active smoker	7 (14)
Non smoker	25 (50)
Ex-smoker	18 (36)
Alcohol history, *n* (%)	
Yes	12 (24)
No	38 (76)
Family history, *n* (%)	
Yes	14 (28)
No	36 (72)
Tumor location, *n* (%)	
Tongue	7 (14)
Mouth	6 (12)
Salivary gland	3 (6)
Tonsil	2 (4)
Oropharynx	2 (2)
Nasopharynx	26 (52)
Sinuses	1 (2)
Larynx	3 (6)
Stage of tumor, *n* (%)	
1–2	8 (6)
3–4	42 (84)
Type of treatment, *n* (%)	
Radiotherapy	17 (34)
Chemoradiotherapy	33 (66)

IQR, Inter-quartile range.

**Table 2:** Changes in nutritional status and NIS at baseline, end and post-treatment.

Variables	Baseline (*n* = 50)	End of RT (*n* = 50)	Post-RT (*n* = 45)	X^2^	*p*-Value
Body weight (kg) ^†^	60.24 ± 14.73	57.64 ± 12.97	56.26 ± 12.24	20.668	<0.0001 *
PGSGA score ^¶^	8.72 ± 6.86	25.82 ± 5.34	13.11 ± 7.87	58.47	<0.0001 *
Muscle mass (kg) ^†^	43.03 ± 8.12	41.30 ± 8.13	42.31 ± 1.08	0.708	<0.0001 *
Fat mass (kg) ^¶^	15.23 ± 9.17	12.67 ± 8.3	11.58 ± 8.29	61.1	<0.0001 *
NIS score ^¶^	21.78 ± 4.59	48.34 ± 8.79	27.08 ± 10.19	67.32	<0.0001 *
Total Energy Intake (Kcal/day) ^†^	1412 ± 589	1554 ± 482	1598 ± 563	0.771	0.512
Total protein Intake (g/day) ^†^	63 ± 60	66 ± 21	80.8 ± 48.03	13.1	0.067
Total energy intake/current weight (kcal/kg BW) ^†^	23.60 ± 8.58	26.26 ± 9.33	27 ± 1.7	0.965	0.402
Total protein intake/current weight (g/kg/BW) ^†^	1.03 ± 0.43	1.11 ± 0.36	1.27 ± 0.80	1.723	0.049 *
Oral food Energy Intake (Kcal/kg/day) ^†^	1355 ± 62	413 ± 426	811 ± 548	10.709	<0.0001 *
Oral food protein Intake(g/day) ^†^	60 ± 31	16 ± 21	41.4 ± 30.2	5.881	<0.0001 *
ONS Energy Intake (Kcal/day) ^¶^	56 ± 181	1141 ± 572	1086 ± 569	72.473	<0.0001 *
ONS Protein Intake (g/day) ^¶^	2 ± 8	50 ± 24	48 ± 24	71.605	<0.0001 *

Mean, standard deviation (SD); * Significant (*p* < 0.05) ^†^ Analyzed by General Linear Model repeated measures. A Greenhouse–Geisser correction for degrees of freedom was used because of deviation from sphericity; ^¶^ Analyzed by the Friedman test; a Wilcoxon signed-rank-sum test (baseline vs. end of RT). Abbreviations: PG-SGA: patient-generated subjective global assessment, BMI: body mass index, NIS: nutrition impact symptoms, ONS: oral nutrition supplements.

**Table 3:** Prevalence of ONS use of HNC.

ONS Use *n* (%)	Baseline (*n* = 50)	End of Treatment (*n* = 50)	Post-Treatment (*n* = 45)
Yes	5 (10)	48 (96)	40 (80)
No	45 (90)	2 (4)	5 (10)

Abbreviations: ONS: oral nutrition supplements.

**Table 4:** NIS experiences in HNC.

Nutrition Impact Symptoms *n* (%)	Baseline (*n* = 50)	End of Treatment (*n* = 50)	Post-Treatment (*n* = 45)
Taste changes	5 (10)	50 (100)	38 (76)
Difficulty swallowing	9 (18)	47 (94)	24 (48)
Difficulty chewing	26 (52)	47 (94)	32 (64)
Constipation	8 (16)	29 (58)	11 (22)
Loss of appetite	19 (38)	47 (94)	27 (54)
Dry mouth	19 (38)	50 (100)	36 (72)
Pain	11 (22)	45 (90)	22 (44)
Anxious	9 (18)	22 (44)	8 (16)
Nausea	4 (8)	22 (44)	6 (12)
Lack of Energy	14 (28)	46 (92)	22 (44)
Sore mouth	6 (12)	43 (86)	12 (24)
Diarrhea	0 (0)	4 (8)	1 (2)
Thick saliva	12 (24)	48 (96)	29 (58)
Depressed	2 (4)	10 (20)	8 (16)
Fullness	4 (8)	26 (52)	9 (18)
Vomiting	3 (6)	11 (22)	4 (8)
Smell bothersome	6 (12)	36 (72)	9 (18)

**Table 5:** Nutrition Impact Symptoms (NIS) interference scores from the Head and Neck Symptoms Checklist (HNSC©) of the patients (*n* = 50) at baseline, end and post-treatment.

NIS Interference Score (1–5)	Baseline (*n* =50)	End of Treatment (*n* = 50)	Post-Treatment (*n* = 45)
Median (IQR)			
Taste changes	1 (1–1)	5 (4–5)	2 (2–3)
Difficulty swallowing	1 (1–1)	4 (3–5)	2 (1–2.5)
Difficulty chewing	2 (1–3.25)	4 (3–5)	2 (1–3)
Constipation	1 (1–1)	2 (1–3)	1 (1–1.25)
Loss of appetite	1 (1–2)	5 (4–5)	2 (1–3)
Dry mouth	1 (1–2)	4 (3–5)	2 (2–3)
Pain	1 (1–1)	4 (3–4.25)	1 (1–2)
Anxious	1 (1–1)	1 (1–3)	1 (1–1)
Nausea	1 (1–1)	1 (1–3)	1 (1–1)
Lack of Energy	1 (1–2)	3 (2.75–4)	1 (1–3)
Sore mouth	1 (1–1)	4 (2–5)	1 (1–2)
Diarrhea	1 (1–1)	1 (1–1)	1 (1–1)
Thick saliva	1 (1–1.25)	4 (3–4)	2 (1–2)
Depressed	1 (1–1)	1 (1–1)	1 (1–1)
Fullness	1 (1–1)	2 (1–3)	1 (1–1)
Vomiting	1 (1–1)	1 (1–1)	1 (1–1)
Smell bothersome	1 (1–1)	3 (1–4)	1 (1–1)

Note: Prevalence NIS when severity scores ≥ 2. Mann–Whitney U test.

## Data Availability

Data are available upon request.
